# Fatigue in Multiple Sclerosis: Neural Correlates and the Role of Non-Invasive Brain Stimulation

**DOI:** 10.3389/fncel.2015.00460

**Published:** 2015-11-30

**Authors:** Moussa A. Chalah, Naji Riachi, Rechdi Ahdab, Alain Créange, Jean-Pascal Lefaucheur, Samar S. Ayache

**Affiliations:** ^1^EA 4391, Excitabilité Nerveuse et Thérapeutique, Université Paris-Est-CréteilCréteil, France; ^2^Service de Physiologie – Explorations Fonctionnelles, Hôpital Henri Mondor, Assistance Publique – Hôpitaux de ParisCréteil, France; ^3^Neurology Division, University Medical Center Rizk HospitalBeirut, Lebanon; ^4^Service de Neurologie, Hôpital Henri Mondor, Assistance Publique – Hôpitaux de ParisCréteil, France

**Keywords:** multiple sclerosis, fatigue, tDCS, non-invasive brain stimulation, network

## Abstract

Multiple sclerosis (MS) is a chronic progressive inflammatory disease of the central nervous system (CNS) and the major cause of non-traumatic disability in young adults. Fatigue is a frequent symptom reported by the majority of MS patients during their disease course and drastically affects their quality of life. Despite its significant prevalence and impact, the underlying pathophysiological mechanisms are not well elucidated. MS fatigue is still considered the result of multifactorial and complex constellations, and is commonly classified into “primary” fatigue related to the pathological changes of the disease itself, and “secondary” fatigue attributed to mimicking symptoms, comorbid sleep and mood disorders, and medications side effects. Radiological, physiological, and endocrine data have raised hypotheses regarding the origin of this symptom, some of which have succeeded in identifying an association between MS fatigue and structural or functional abnormalities within various brain networks. Hence, the aim of this work is to reappraise the neural correlates of MS fatigue and to discuss the rationale for the emergent use of noninvasive brain stimulation (NIBS) techniques as potential treatments. This will include a presentation of the various NIBS modalities and a suggestion of their potential mechanisms of action in this context. Specific issues related to the value of transcranial direct current stimulation (tDCS) will be addressed.

## Introduction

Multiple sclerosis (MS) is a chronic progressive inflammatory disease of the central nervous system (CNS) and represents the major cause of non-traumatic disability in young adults (Noseworthy et al., 2000; Compston and Coles, 2008). Its estimated prevalence in Europe is 83 per 100,000, with rates being approximately twice as high for women as for men and lower in the southern than in the northern European countries (Pugliatti et al., 2006).

The precise etiology of MS remains obscure and involves a plethora of mechanisms. Its clinical course is highly heterogeneous, during which patients may develop various motor, sensory, cognitive, and behavioral symptoms. The available treatments consist of the disease-modifying therapies (such as the immunosuppressants, immunomodulators, and monoclonal antibodies) (Wingerchuk and Carter, 2014); in addition to several symptomatic treatments that are usually prescribed to control disorders of strength, sensation, mood, sleep, etc.

Among the frequently encountered symptoms in MS, fatigue remains the most challenging one, majorly altering the quality of life (Krupp et al., 1988; Janardhan and Bakshi, 2002; Merkelbach et al., 2002; Giovannoni, 2006; Induruwa et al., 2012). Indeed, it affects up to 75% of MS patients at some point in their disease course (Bakshi et al., 2000; Lerdal et al., 2007; Kos et al., 2008), gets exacerbated during the day (Comi et al., 2001; Morris et al., 2002; Mills and Young, 2008; Krupp et al., 2010), and increases with hot and humid environment (Bakshi, 2003; Bol et al., 2012; Leavitt et al., 2012).

Although fatigue is reported by the majority of individuals as their most common and disabling symptom (Krupp et al., 1989, 2010; Fisk et al., 1994a,b; Colosimo et al., 1995; Bakshi, 2003; Lobentanz et al., 2004; Flensner et al., 2008), no clear definition exists in the literature; which makes it at a time hard to be described by patients and diagnosed by physicians. Such a difficulty is further exacerbated by the diversity of the fatigue assessment tools employed by scientists and researchers. In the majority of cases, fatigue is self-reported using various subjective scales, like Fatigue Severity Scale (FSS) or Modified Fatigue Impact Scale (MFIS), among others.

From an etiological perspective, no single mechanism accounts for its occurrence, and MS fatigue is rather seen as a complex and multifactorial constellation (Bakshi, 2003; MacAllister and Krupp, 2005; Kos et al., 2008; Braley and Chervin, 2010; Krupp et al., 2010; Vucic et al., 2010; Induruwa et al., 2012). In an attempt to account for its origin, many hypotheses have been raised and include the processes of demyelination and axonal loss that involve various cortical and subcortical regions; the neuroimmune dysregulation supported by the elevated levels of cytokines and other inflammatory mediators encountered in MS patients; and the neuroendocrine dysfunction along the hypothalamo-pituitary-adrenal (HPA) axis, among others.

Facing this reality, various pharmacological (dopaminergic drugs, psychostimulants, etc.) and alternative interventions (aerobic exercises, cooling therapies, psychotherapies, etc.) have been tried but only resulted in limited benefits (Vucic et al., 2010; Induruwa et al., 2012). Nowadays, non-invasive brain stimulation (NIBS) techniques have gained an important interest in the treatment of many neuropsychiatric disorders; hence, they might be of some help in terms of MS fatigue.

In this review, we will first revisit the definition of this symptom, summarize its various types, and shed the light on the central fatigue encountered in MS patients; a particular emphasis will be given to the available data regarding the proposed fatigue networks. A final part will be dedicated to the NIBS techniques and their possible mechanisms in this context. The pharmacological and other alternative therapies are beyond the scope of this work (For reviews see Vucic et al., 2010; Induruwa et al., 2012).

## Multiple sclerosis fatigue

To start, the definition of fatigue differs widely among patients, researchers, and physicians. From the patients' perspective, fatigue is usually reported as “excessive tiredness,” “malaise,” or “weakness” (Krupp, 2003). As for researchers and physicians, the definition varied among the available data. For instance, Wessely observed the fatigue in terms of its origin and referred to it as having its source at the level of the upper motor neuron or above (Wessely, 1998). Gandevia et al. defined it as a progressive exercise-induced reduction in voluntary activation of a muscle (Gandevia, 2001). Chaudhuri and Behan considered it a failure to initiate and/or sustain attention tasks and physical activities requiring self-motivation in the absence or not related to physical or cognitive dysfunction (Chaudhuri and Behan, 2000). Other authors described it as a subjective lack of physical and/or mental energy (Benedict et al., 2005). Interestingly, a medical definition of fatigue was proposed in one study which consisted of a qualitative phase followed by a cross-sectional questionnaire survey in 40 MS patients (Mills and Young, 2008). Here, the symptom was defined as a reversible motor and cognitive impairment, with a reduced motivation and an increased desire to rest. It can occur either spontaneously or following various factors such as mental or physical activity, humidity, infections, and food ingestion.

Second, fatigue encountered in MS patients is commonly classified into a “primary” fatigue related to the CNS pathological changes in the context of the disease itself; and a “secondary” fatigue attributed to MS-related comorbidities. This includes weakness that can mimic fatigue (van der Werf et al., 1998), mood and sleep disorders (Möller et al., 1994; Induruwa et al., 2012), and medications side effects (Braley and Chervin, 2010). It is noteworthy that disease-modifying therapies themselves could have some impact on MS fatigue. For instance, MS patients treated with Interferon-beta can experience fatigue (Braley and Chervin, 2010). Conversely, the monoclonal antibody Natalizumab could reduce fatigue, as shown in TYNERGY trial (For reviews see Hoepner et al., 2014).

Primary fatigue results from a spectrum where one pole is the inability to generate the force required to perform the task due to a failure of force production at the muscle level (“peripheral fatigue”); and the other pole is the inability to sustain the required neural drive to muscle because of supraspinal, spinal, and even peripheral nerve contribution (“central fatigue”) (Gandevia, 2001; Zwarts et al., 2008). Interestingly, “central fatigue” can be the result of both cognitive and physical exertion (Claros-Salinas et al., 2013) and can reflect either a subjective sensation (fatigue) or an objective change in performance (fatigability) (Kluger et al., 2013).

Another way to perceive MS fatigue consists of differentiating “trait” from “state” fatigue. The “trait” fatigue was the focus of several papers and was defined as a stable state that expresses the global status of patients, which does not significantly change over time, and usually assessed with fatigue scales (Sepulcre et al., 2009; Calabrese et al., 2010; Genova et al., 2013). In contrast, “state” fatigue was the subject of few works only; it is a transient condition characterized by a decreased performance during an acute but sustained effort (DeLuca et al., 2008; Genova et al., 2013). In addition, it can fluctuate in response to external and internal factors, and is usually tested during task performance.

## MS fatigue, disability, and disease duration

Most of the studies that investigated MS fatigue aimed to understand the contributory role of clinical, demographic and neuroimaging data. In this perspective, conflicting data were found regarding the relationship between fatigue severity and the level of physical disability. Although many studies have established a positive correlation between fatigue scores and the degree of physical disability based on the Expanded Disability Status Scale (EDSS) (Colosimo et al., 1995; van der Werf et al., 1998; Flachenecker et al., 2002; Niepel et al., 2006; Pellicano et al., 2010), others have either found a weak association (Ford et al., 1998; Pittion-Vouyovitch et al., 2006; Yaldizli et al., 2011), or failed to detect any relationship (Krupp et al., 1988; Roelcke et al., 1997; Sheean et al., 1997; Provinciali et al., 1999; Rocca et al., 2009; Cruz Gómez et al., 2013; Derache et al., 2013; Gobbi et al., 2014a; Hesse et al., 2014). Such findings might be related to the multi-component aspect of this symptom and/or the differences among these studies in terms of sample size, recruited MS subtypes, EDSS cut-offs, and the subjective tools used for reporting fatigue. Furthermore, the majority of the studies could not correlate fatigue scores with disease duration (Roelcke et al., 1997; Bakshi et al., 1999; Niepel et al., 2006; Pellicano et al., 2010; Gold et al., 2011; Yaldizli et al., 2011; Derache et al., 2013).

Admitting this apparent occurrence of fatigue independently from disease duration and disability progression, there is a growing interest in understanding the radiological and neurophysiological underpinnings of MS fatigue.

## Neural correlates of MS fatigue

### Impact of white matter and gray matter abnormalities

Magnetic resonance imaging (MRI) studies revealed controversial data regarding the relationship between fatigue severity and each of the brain atrophy and lesion load. While some studies of them have yielded positive associations, others failed to detect any relationship. The latter finding may be attributed in part to the small sample size selected in some of these studies, and to the cross-sectional or short-term longitudinal follow up design adopted by most of them. A summary is available in Table 1.

**Table 1:** **Studies evaluating the role of gray and white matter abnormalities in multiple sclerosis fatigue**.

**Authors (Year)**	**Technique**	**Parameter (s)**	**Scale**	**Population**	**Correlation with fatigue**
van der Werf et al., 1998	MRI	Lesion load	CIS-Fatigue scale	45 MS	No
		Brain atrophy			
Bakshi et al., 1999	MRI	Lesion load	FSS	71 MS	No
		Brain atrophy			
Mainero et al., 1999	MRI	Gd-enhancing lesions	FSS	11 MS	No
Codella et al., 2002a	MRI (MTI, DTI)	Quantification of lesions and NABT	FSS	28 MS	No
Tartaglia et al., 2004	MRS	Axonal damage	FSS	73 MS	Yes
Marrie et al., 2005	BPF	Brain atrophy	SIPSR	134 MS	Yes
Tedeschi et al., 2007	MRI	Lesion load	FSS	222 MS	Yes
		WM and GM atrophy			
Papadopoulou et al., 2013	MRI	GM volume	FSMC	91 MS	No
		Cortical volume			
		WM lesions			
Gobbi et al., 2014a	MRI	GM and WM atrophy	FSS	123 MS	No
		Lesions distribution			

*BPF, brain parenchymal fraction; CIS-FATIGUE, Checklist Individual Strength -fatigue questionnaire; DTI, Diffusion tensor imaging; FSMC, Fatigue Scale for Motor and Cognitive Functions; GM, Gray matter; MRI, magnetic resonance imaging; MRS, Magnetic Resonance Spectroscopy; MS, Multiple sclerosis; MTI, Magnetization transfer imaging; NABT, normally appearing brain tissues; SIPSR, Sickness Impact Profile's Sleep and Rest Scale; WM, white matter*.

Interestingly, a large number of works have succeeded in establishing a link between fatigue scores and gray matter (GM) and white matter (WM) abnormalities in specific cortical and subcortical regions that were proposed to be major components of the MS fatigue networks. Such networks will constitute the main scope of this section.

### Anatomical correlates of MS fatigue

#### The fronto-striatal network

Among the early evidences of cortico-subcortical circuit dysfunction as a substrate for MS fatigue, the involvement of a “fronto-striatal network” was highlighted by two studies. In the first one, fatigued MS (F-MS) patients had a predominant reduced cerebral metabolic rate of glucose in the frontal lobe and basal ganglia (Roelcke et al., 1997); FSS scores were inversely correlated with such a rate in the right dorsolateral prefrontal cortex (DLPFC). In line with these results, Pardini et al. reported a significant association between MFIS scores and a cluster of voxels located in the left frontal WM, which was further found to be involved in the fronto-striatal network, among others (Pardini et al., 2010). In a third study, MS patients underwent functional MRI (fMRI) during the performance of a single motor task using the right hand (Specogna et al., 2012). Compared to non-fatigued MS (NF-MS) patients, F-MS patients had a greater activation of the premotor area ipsilateral to the movement, the right putamen, and the right DLPFC. Conversely, Codella et al. failed to demonstrate a difference in fronto-striatal GM pathology between F-MS and NF-MS patients, or a correlation between such findings and FSS scores. These results could be partially attributed to the small sample size (Codella et al., 2002b). Table 2 provides a summary of the abovementioned studies.

**Table 2:** **Studies evaluating the role of fronto-striatal networks in multiple sclerosis fatigue**.

**Authors (Year)**	**Technique**	**Scales**	**Population**	**Outcome**
Roelcke et al., 1997	PET	FSS	35 MS	Significant correlation between FSS and glucose reduction in the right DLPFC
Pardini et al., 2010	MRI (DTI)	MFIS	40 RRMS	Significant correlation between MFIS scores and deep left frontal WM, a region found to be involved in fronto-striatal networks, among others
Specogna et al., 2012	fMRI (+single motor task)	FSS	24 RRMS	Significant correlation between FSS scores and activation patterns of the premotor area

*DTI, diffusion tensor imaging; FSS, Fatigue Severity Scale; fMRI, functional magnetic reasoning imaging; GM, gray matter; MFIS, Modified Fatigue Impact Scale; MTI, magnetization transfer imaging; PET, Positron Emission Tomography; PMC, premotor cortex; RRMS, relapsing remitting multiple sclerosis; WM, white matter; SMA, supplementary motor area*.

#### The parieto-striatal network

A parieto-striatal network was the object of one MRI study involving 15 F-MS and 15 NF-MS patients. A significant association was found between fatigue scores and the lesion load in the parietal lobe, internal capsule, and periventricular trigone, emphasizing the role of this network in MS fatigue (Colombo et al., 2000).

#### Deep gray matter substrates

A group of authors correlated MS fatigue with isolated deep GM pathologies. One study assessed the involvement of caudate, putamen, and thalamus based on the mean T1 relaxation time (Niepel et al., 2006), an MRI parameter previously found to correlate with the degree of disability in MS patients (Parry et al., 2002). Compared to their non-fatigued counterparts, F-MS patients had significantly higher median T1 values in the thalami, which were significantly correlated to FSS scores. The thalamic nuclei represent an essential relay center in information integration, the dysfunction of which might contribute to fatigue. Another work employed the proton magnetic resonance spectroscopy to study the relationship between fatigue scores and the N-acetyl aspartate to creatinine ratio (NAA/cr), a neural marker of axonal damage, within the frontal cortex and basal ganglia (Téllez et al., 2008). A correlation was only found between the physical domain of MFIS and NAA/cr within the lentiform nucleus complex. A third study assessed the perfusion parameters of deep GM and MS fatigue based on the multidimensional fatigue inventory (MFI) (Inglese et al., 2007). An association was found between MFI scores and the cerebral blood volume and flow in the thalamus and basal ganglia (for a summary, see Table 3).

**Table 3:** **Studies supporting the role of deep gray matter substrates in multiple sclerosis fatigue**.

**Authors (Year)**	**Technique**	**Scales**	**Population**	**Outcome**
Niepel et al., 2006	MRI	FSS	52 RRMS	Correlation of FSS with abnormal T1 relaxation time within the thalami
Inglese et al., 2007	MRI	MFI	22 MS (11RRMS, 11 PPMS)	Correlation of MFI with the cerebral blood volume and flow in the thalamus and basal ganglia
Téllez et al., 2008	^1^H-MRS	MFIS, FSS	41 RRMS	Correlation of physical domain of MFIS with abnormal NAA/cr ratio within the lentiform nucleus

*FSS, Fatigue Severity Scale; ^1^H-MRS, proton magnetic reasoning imaging; MFI, Multidimensional Fatigue Inventory; MFIS, Modified Fatigue Impact Scale; NAA/cr, N-acetyl aspartate to creatinine ratio; RRMS, relapsing remitting multiple sclerosis; PPMS, primary progressive MS*.

It is important to note that the difference in the fatigue scales among these three studies might be behind the minor variation in the results. In fact, the 7-item FSS has some limitations due to its classic ordinal construct (Krupp et al., 1988); the 21-item MFIS is a modified version of the original fatigue impact scale (FIS) that explores the physical, psychosocial and cognitive domains of fatigue (Fisk et al., 1994a,b); and the 20-item MFI employed in the last study evaluates five fatigue dimensions, namely the general, physical and mental fatigue along with the reduced motivation and activity (Smets et al., 1995).

#### Cortico-cortical networks

Among the works dedicated to MS fatigue, some attention was paid on the pathological changes affecting the cortico-cortical networks, particularly those involving the frontal and parietal cortices. In this setting, six studies were found to fulfill these criteria. In one study, electroencephalography (EEG) recording were performed during and following the performance of a simple motor task (Leocani et al., 2001). Compared to NF-MS patients and healthy controls, F-MS patients had higher event-related desynchronization (ERD) over the midline frontal structures during the movement and lower event-related synchronization (ERS) in the contralateral central area after the movement (Leocani et al., 2001). These are the respective markers of cortical activation and idling. Therefore, this altered balance between excitation (ERD) and inhibition (ERS) might result from axonal damage and the subsequent loss of functional intra-cortical connections. The post-movement loss of inhibition (ERS) seems to trigger brain activation and might account for MS fatigue. Tartaglia et al. found that, during the performance of a fatiguing cognitive task, MS patients exhibited an altered pattern of activation within the left primary sensory and premotor cortices and the supplementary motor area, despite the lack of performance difference in comparison to the healthy group (Tartaglia et al., 2008). In another study, fatigue was significantly correlated with frontal GM atrophy especially involving the superior frontal gyrus (SFG) on the left and the middle frontal gyrus (MFG) bilaterally, and with lesion load namely found within the left frontal and right parieto-temporal areas (Sepulcre et al., 2009). In addition, using DTI-based tractography, one study established a correlation between fatigue scores and a significant cluster of voxels in the deep WM involving fronto-frontal fibers, among others (Pardini et al., 2010). It is noteworthy that fatigue scores were frequently found to correlate with isolated regional metabolic changes in the posterior parietal cortex (PPC) (Pellicano et al., 2010), left precentral gyrus (Riccitelli et al., 2011), bilateral frontal cortices (Huolman et al., 2011), left superior frontal and right inferior temporal gyri (Rocca et al., 2014), and left supplementary motor area (Cruz Gómez et al., 2013). Other studies reported an association between fatigue and abnormalities involving the corpus callosum and its radiating fibers that primarily ensure the interhemispheric cortico-cortical connections (Yaldizli et al., 2011, 2014; Gobbi et al., 2014b; Rocca et al., 2014). The above-mentioned papers are summarized in Table 4.

**Table 4:** **Studies highlighting the impact of cortico-cortical connections in multiple sclerosis fatigue**.

**Authors (Year)**	**Technique**	**Scales**	**Population**	**Outcome**
Leocani et al., 2001	EEG (during simple motor task)	FSS	23 MS	Correlation of fatigue scores with ERD over the midline frontal structures during movement, and an inverse correlation of those scores with contralateral central ERS post-movement
Tartaglia et al., 2008	fMRI (during PASAT)	FSS	10 RRMS	Correlation between fatigue scores and an altered activation pattern within sensory and motor areas
Sepulcre et al., 2009	MRI	MFIS	60 MS	Correlation between fatigue scores and fronto-parietal pathologies
Pardini et al., 2010	MRI	MFIS	40 RRMS	Correlation between MFIS scores and deep left frontal WM, a region found to be involved in fronto-frontal and fronto-occipital networks
Pellicano et al., 2010	MRI	MFIS	20 RRMS	Correlation of fatigue scores with posterior parietal cortical thickness
			4 SPMS	
Yaldizli et al., 2011	MRI	FSS	70 RRMS	Association between fatigue scores and CC atrophy
Huolman et al., 2011	fMRI (during mPVSAT)	–	15 MS	Hyperactivation of bilateral frontal regions
Riccitelli et al., 2011	MRI	FSS	24 RRMS	Correlation between fatigue scores and GM atrophy within the left precentral gyrus
Cruz Gómez et al., 2013	fMRI	FSS	60 RRMS	Correlation between fatigue scores and WM atrophy within the left supplementary motor areas
Rocca et al., 2014	MRI	FSS	63 MS	Correlations between fatigue scores and atrophy of the right inferior temporal gyrus, left superior frontal gyrus, and forceps major
Yaldizli et al., 2014	MRI	FSS	113 MS	Association between fatigue scores and CC atrophy
Gobbi et al., 2014b	MRI	MFIS	147 MS	Association between fatigue and CC pathology

*CC, Corpus callosum; EEG, electroencephalography; ERD, event related desynchronization; ERS, event related synchronization; F, fatigued; FSS, Fatigue Severity Scale, fMRI, functional magnetic resonance imaging, GM, gray matter; HCs, Healthy controls; MFIS, Modified Fatigue Impact Scale; mPVSAT, modified Paced Visual Serial Addition Test; PASAT, Paced Auditory Serial Addition Test; RRMS, relapsing remitting multiple sclerosis; SPMS, secondary progressive multiple sclerosis; WM, white matter*.

#### The cortico-striato-thalamo-cortical loop

Considering this bunch of evidence involving various brain areas and admitting the heterogeneous nature of MS, a large neural network should be considered in the context of MS fatigue.

First, using morphological MRI, one study has found that F-MS patients had atrophy in the striatum, thalamus, SFG, and intraparietal gyrus (IPG). Correlations were observed between MFIS cognitive subscale and each of the striatal volume, and the cortical thickness of the PPC and the MFG. The MFIS physical subscale was also correlated with the striatal volume and SFG cortical thickness (Calabrese et al., 2010). Andreasen et al. used DTI and magnetization transfer imaging to study the central motor drive during isometric contraction (Andreasen et al., 2010). They found that F-MS patients had regional atrophy in the DLPFC, PPC, and basal ganglia. However, no correlation was found between these atrophies and FSS scores, a finding that might be attributed to the small sample.

Data from fMRI studies have documented an altered pattern of regional activation and functional connectivity (FC) in F-MS patients at rest or during the performance of fatiguing motor and cognitive tasks. In one study, F-MS patients showed hypoactivation within the ipsilateral rolandic operculum and precuneus, as well as the contralateral thalamus and MFG. FSS scores were inversely correlated with the activation pattern of the contralateral thalamus and the ispsilateral rolandic operculum (Filippi et al., 2002). A second work compared the brain activation pattern of fatigable and non-fatigable MS patients following interferon injection (Rocca et al., 2007). Following the injection, fatigable patients showed increased activation of the basal ganglia, thalami, primary sensorimotor cortex, supplementary motor area, and several frontal regions. In a third study, kinematic and fMRI data were obtained from healthy controls, F-MS and NF-MS patients. Compared to the other groups, F-MS patients had an increased activation of the left secondary somatosensory cortex and the right precuneus and decreased activation of the right thalamus, right basal ganglia, left inferior frontal gyrus (IFG) during in-phase and anti-phase movements (Rocca et al., 2009). Here, the fatigue effect was mainly located in fronto-parietal regions including the left IFG, and left postcentral gyrus, among others. A fourth study found an association between mental fatigue and hyperactivation in the fronto-parietal regions, basal ganglia, and thalamus (DeLuca et al., 2008).

It is worth noting that two other works focused on the brain activation patterns and FC during the performance of a mentally fatiguing cognitive task. In the first study, MS patients had stronger cortico-cortical and subcortico-subcortical FC and weaker cortico-subcortical FC between the left PPC and the right caudate head compared to healthy controls (Engström et al., 2013). Here, MS fatigue was significantly correlated with the right substantia nigra (SN) activation and marginally with the left PPC activation; the reported activation pattern within the SN might have increased the thalamic inhibition and accounted for MS fatigue. In the other study, MS patients had hyperactivation of the caudate nucleus which was associated with state fatigue (Genova et al., 2013). In the same work, the authors used DTI and demonstrated a correlation between FSS scores and reduced fraction anisotropy (FA) in the anterior internal capsule, a structure having connections with the caudate nucleus and the thalamus. These findings support the role of the “striatal-thalamic-frontal” system in MS fatigue (Genova et al., 2013).

In addition, Derache et al. found that their F-MS cohort had a significant reduction of GM density in clusters, some of which are located in the bilateral frontal cortex and in the left parietal cortex. Fatigue scores were negatively correlated with each of GM density in the same involved fronto-parietal areas, the GM density in bilateral thalamus and the rest central glucose metabolic rate within the basal ganglia (Derache et al., 2013). Finally, in a resting-state (rs) fMRI study conducted by Finke et al. fatigue severity was negatively correlated with the FC between the basal ganglia and the medial prefrontal gyrus (PFG), precuneus, and posterior cingulate cortex; and positively with the FC between the caudate nucleus and the motor cortex (Finke et al., 2014).

To sum up, these studies altogether support the existence of a cortico-striato-thalamo-cortical loop as a neural correlate of MS fatigue. The latter seems to englobe motor and non-motor circuits acting together to induce the symptom in question (Table 5).

**Table 5:** **Studies highlighting the role of the cortico-striato-thalamo-cortical loop of fatigue in multiple sclerosis**.

**Authors (Year)**	**Technique**	**Scales**	**Population**	**Outcome**
Filippi et al., 2002	fMRI (during simple motor task)	FSS	29 MS	Inverse correlation of FSS scores with the activation pattern of the contralateral thalamus and the ipsilateral rolandic operculum
Rocca et al., 2007	fMRI (during simple motor task, before and after IFN injection)	FSS	22 MS	Post-injection fatigable patients showed increased activations of the basal ganglia, thalami, primary sensorimotor cortex, SMA, and several frontal regions
DeLuca et al., 2008	fMRI (during mSDMT)	N/A	12 MS	Association between mental fatigue and hyperactivation within the fronto-parietal regions, basal ganglia, and thalamus
Rocca et al., 2009	MRI, fMRI (during kinematic movement)	FSS	24 RRMS	Association between fatigue and the activation pattern of the fronto-parietal regions including the left IFG and left postcentral gyrus, among others
Andreasen et al., 2010	MRI (during isometric contraction)	FSS	34 MS	No correlation between FSS scores and the atrophy found within the DLPFC, PPC, and basal ganglia
Calabrese et al., 2010	MRI	FSS/MFIS	152 RRMS	Correlation of cognitive MFIS scores with striatal volume and the cortical thickness of PPC and MFG
				Correlation of physical MFIS scores with striatal volume and the cortical thickness of SFG
Engström et al., 2013	fMRI (during a complex cognitive task)	FIS	15 MS	Correlation of fatigue with right substansia nigra hyperactivation
Derache et al., 2013	PET/MRI	MFIS	17 RRMS	Negative correlation between fatigue scores and clusters of reduced GM density within the fronto-parietal cortices and the thalami; and with the rest cerebral glucose metabolic rate of the basal ganglia
Genova et al., 2013	fMRI (during a sustained cognitive task)	VAS	12 MS	Association of state fatigue with caudate nucleus hyperactivation
	MRI (DTI)	FSS	13 MS	Correlation between FSS scores and the reduced fraction anisotropy in the anterior internal capsule
Finke et al., 2014	MRI, fMRI	FSS	44 RRMS	Negative correlation between fatigue severity and rc-FC of basal ganglia and medial PFG, precuneus, posterior cingulate cortex
				Positive correlation between fatigue severity and rs-FC of caudate and motor cortex

*DLPFC, dorsolateral prefrontal cortex; FSS, Fatigue Severity Scale; F, fatigued; FIS fatigue impact scale; fMRI, functional magnetic resonance imaging; HCs, Healthy controls; IFN, interferon; IPG, intraparietal gyrus; L, left; rs-FC, resting state functional connectivity; MFG, middle frontal gyrus; MFIS, Modified Fatigue Impact Scale; mSDMT, modified Single Digit Modalities Test; N/A, Not applicable; PET, Positron Emission Tomography; PFG, prefrontal gyrus; PPC, posterior parietal cortex; R, right; RRMS, relapsing remitting multiple sclerosis; SFG, superior frontal gyrus; SMA, supplementary motor area; VAS, Visual Analog Scale*.

#### Other neural correlates

Apart from the above-discussed circuits, a number of reports have linked MS fatigue to other cerebral structures. For instance, one study has found an association between MS fatigue and an altered integrity of the fibers that connect the posterior hypothalamus and the mesencephalon (Hanken et al., 2015). This was in line with two previous works. In the first one, F-MS patients exhibited a higher activity of the HPA axis than those without fatigue (Gottschalk et al., 2005). As for the second one, FSS scores were positively correlated with the T1 relaxation time values within the hypothalamus of MS patients (Zellini et al., 2009).

To note, other neural correlates were associated with fatigue, and include the cerebellum (Roelcke et al., 1997; Filippi et al., 2002; Rocca et al., 2009), the cingulate cortex (Roelcke et al., 1997; Filippi et al., 2002; Rocca et al., 2007; Tartaglia et al., 2008; Sepulcre et al., 2009; Andreasen et al., 2010; Finke et al., 2014; Pardini et al., 2015), the right anterior thalamic radiations (Bester et al., 2013; Gobbi et al., 2014b; Rocca et al., 2014), and the nucleus accumbens (Rocca et al., 2014), among others.

### Neuro-immune correlates of MS fatigue

Immunological factors have long been recognized as key factors in the pathophysiology of MS (Rovaris et al., 1996; Khademi et al., 2000; Baraczka et al., 2003). Hence, several studies have looked at the potential role of the inflammatory cytokines in MS fatigue. Interestingly, a significant correlation was found between the level of response to Amantadine and Pemoline -pharmacological treatments of fatigue- and the reduction in serum levels of interleukin 1-beta (IL-1β) and interleukin 6 (IL-6) in MS patients (Bertolone et al., 1993). Furthermore, the median levels of tumor necrosis factor alpha (TNF-α) mRNA expression and the serum levels of TNF-α and interferon gamma (IFN-γ) were found to be significantly higher in F-MS patients than in NF ones (Flachenecker et al., 2004; Heesen et al., 2006; Pokryszko-Dragan et al., 2012). Additionally, a positive relationship was found between IL-6 serum levels and fatigue scores in MS patients (Malekzadeh et al., 2015). Moreover, a higher frequency of cytokine producing CD8+ T cells was found among MS patients and was the only significant predictor of fatigue scores (Gold et al., 2011).

All these studies hint toward the role of inflammation in inducing MS fatigue, which might be of potential interest for future targeted therapies.

## NIBS and MS fatigue

Various pharmacological and non-pharmacological interventions have been tried to treat MS fatigue, but showed limited benefits and multiple side effects (Induruwa et al., 2012; Khan et al., 2014). As discussed above, neuroimaging studies have focused on the neural network changes underlying MS fatigue. Hence, acting on these networks could be of particular help in the management of such a debilitating symptom, and thereby would support the use of NIBS in this context.

### NIBS principles

There is a growing interest in studying the therapeutic effects of NIBS techniques in neurological or psychiatric diseases (Lefaucheur et al., 2014). These techniques include repetitive transcranial magnetic stimulation (rTMS) and transcranial direct current stimulation (tDCS).

rTMS consists of a transcranial delivery of an electromagnetic field via a stimulation coil localized on the patient's scalp. This results in an intracortical current that is strong enough to trigger action potentials (APs) (Lefaucheur, 2012). rTMS mainly acts by modifying the cortical excitability, which majorly depends on the stimulation frequency. It was shown that low frequencies (< 1 Hz) and high frequencies (>5 Hz) have respectively inhibitory and excitatory effects (Pascual-Leone et al., 1994). Over the last few years, rTMS has gained special interest in the management of MS symptoms. For instance, rTMS over the motor cortex was shown to provide beneficial effects on spasticity (Centonze et al., 2007a), lower urinary tract (LUT) symptoms (Centonze et al., 2007b), and hand dexterity (Koch et al., 2008) in MS patients. Furthermore, intermittent theta burst stimulation (iTBS) -a variant of TMS- ameliorated MS spasticity (Mori et al., 2010a,b) and primed the exercise effect in MS patients (Mori et al., 2011).

As for tDCS, the idea of using electrical current stimulation to modulate brain function dates back to more than 200 years (Priori, 2003; Zago et al., 2008). Data from animal studies revealed that weak direct currents delivered by intracerebral or epidural electrodes were able to modify cortical excitability, an effect that remains stable long after the end of stimulation. In the last few decades, tDCS showed its reliability in changing human cortical function by inducing focal, prolonged yet reversible shifts in cortical excitability (Priori et al., 1998; Nitsche and Paulus, 2000, 2001; Nitsche et al., 2003; Priori, 2003). The technique consists of localizing two electrodes over the scalp, an anode and a cathode, in order to act on various neural circuits. The exposed tissue is polarized and tDCS modifies spontaneous neuronal excitability by a tonic depolarization or hyperpolarization of resting membrane potential, obtained respectively by performing anodal or cathodal tDCS (Creutzfeldt et al., 1962; Purpura and McMurtry, 1965; Nitsche and Paulus, 2011; Paulus et al., 2013; Filmer et al., 2014). tDCS effects depend on several parameters including the electrodes size, type, polarity, and position as well as the current strength and shape, and the stimulation duration (Purpura and McMurtry, 1965; Nitsche and Fregni, 2007; Nitsche et al., 2008).

Moreover, tDCS appears to be an NIBS technique with a good safety profile, easy implementation, good patients' tolerance and little or no adverse effects (Poreisz et al., 2007; Nitsche et al., 2008; Brunoni et al., 2012). In addition, compared to rTMS, placebo-controlled studies are easier to be performed with tDCS since real and sham stimulations are quite indistinguishable (Gandiga et al., 2006). For all these reasons, tDCS would be an important therapeutic modality for cortical dysfunction (Been et al., 2007) constituting an interesting option in the management of MS symptoms, notably fatigue.

Before reviewing NIBS data in terms of MS fatigue, we will present the various aspects that support the specific interest of tDCS use to treat MS-related brain dysfunction.

### Specific interests of tDCS in the management of MS-related disorders

#### tDCS and brain functional connectivity

The modulatory effects of tDCS in terms of the rs-FC of various networks have been supported by numerous fMRI and MRS studies. For instance, anodal tDCS over the left DLPFC increased its connectivity with the right hemisphere (Park et al., 2013) and modified the rs-FC in the fronto-parietal networks (Keeser et al., 2011). In addition, the tDCS effects on brain networks connectivity were demonstrated following motor cortex, left and right inferior frontal gyri (IFG) stimulation. Anodal stimulation of the former would lead to FC changes some of which occur within the cortico-striatal and thalamo-cortical circuits (Polanía et al., 2011a,b, 2012a,b; Sehm et al., 2012, 2013; Amadi et al., 2013; Khan et al., 2013; Lindenberg et al., 2013). Furthermore, acting on the left or right IFG had an important impact on the rs-FC of the language network (Meinzer et al., 2012, 2013; Rosso et al., 2014).

Therefore, based on these data, tDCS might be beneficial in modulating MS-fatigue networks and restoring brain FC through several potential mechanisms.

#### tDCS, conduction failure, and axonal degeneration

Major changes in axonal conduction occur in MS as a result of the inflammatory demyelinating processes and the oxidative stress. First, among the inflammatory mediators, nitric oxide can cause a depolarizing conduction block (Redford et al., 1997; Shrager et al., 1998) which may occur through the activation of persistent Na^+^ channels and the subsequent increase in intracellular Na^+^; or by causing a mitochondrial dysfunction and axonal ATP depletion (Bolanos et al., 1997). The latter can explain the documented Na^+^/K^+^ ATPase failure (Dutta et al., 2006; Waxman, 2006; Mahad et al., 2008) and the reverse action of Na^+^–Ca^2+^ exchanger, leading to intraaxonal calcium level increase, activation of Ca^2+^-dependent apoptosis and axonal degeneration (Bechtold and Smith, 2005; Stys, 2005; Gonsette, 2008). Interestingly, one study has shown that tDCS might act on these mechanisms by modulating the activity of Na^+^ and Ca^2+^ channels (Nitsche et al., 2003). Furthermore, in the rat model of Parkinson disease, tDCS was able to reduce the oxidative stress, which resulted in a cognitive improvement (Lu et al., 2015). Hence, anodal tDCS might enhance axonal conduction along the demyelinated segments through a subthreshold polarizing effect. This might be used to improve MS fatigue.

Beyond demyelination, MS can lead to axonal degeneration, associated with irreversible deficits. Notably, two recent animal studies have documented a positive effect of tDCS on the activation and migration of neural stem cells (Rueger et al., 2012; Keuters et al., 2015). Therefore, tDCS could be helpful in promoting the regeneration processes and ameliorating various MS symptoms in the course of the disease.

#### tDCS and inflammation-induced synaptopathy

Inflammation-induced synaptopathy is the link between inflammation and neurodegeneration, as shown in the animal model of MS. Inflammation might limit the plastic compensatory protective changes in neural networks, which can be the route by which CNS confined inflammation shifts into irreversible neurodegenerative disorders (Centonze et al., 2010). Glutamate is a major excitatory neurotransmitter of the CNS, importantly involved in MS neurodegenerative damage (Srinivasan et al., 2005; Cianfoni et al., 2007). Altered glutamate metabolism and the subsequent abnormal intracellular ion accumulation can lead to excitotoxic neuronal damage (Forder and Tymianski, 2009). MS patients were found to have increased CSF levels of glutamate (Stover et al., 1997; Sarchielli et al., 2003; Srinivasan et al., 2005; Cianfoni et al., 2007), upregulation of glutamate receptors (Newcombe et al., 2008), and altered expression of glutamate transporters (Geurts et al., 2003, 2005; Vallejo-Illarramendi et al., 2006).

In addition, inflammation-dependent activation of the microglia is associated with the release of TNF-α, IFN-γ, and IL-1β (Block and Hong, 2005; Kawanokuchi et al., 2006; Schwartz et al., 2006; Muzio et al., 2007), which can enhance the excitatory synaptic transmission (Stellwagen et al., 2005; Lai et al., 2006; Mizuno et al., 2008), and downregulate the inhibitory Gamma Amino-Butyric acid (GABA) transmission (Stellwagen et al., 2005). These facts are compatible with the case of MS patients who are known to have increased CSF levels of TNF-α, IFN-γ, and IL-1β, among other inflammatory mediators (Rovaris et al., 1996; Khademi et al., 2000; Baraczka et al., 2003).

These findings altogether may explain the inflammation-induced synaptic alterations and the subsequent neurodegenerative processes in MS, and might in turn account for the appearance of MS fatigue.

Knowing the interaction between tDCS and long-term changes in neurotransmission, tDCS effects might occur through the modulation of inflammation-induced synaptopathy in MS patients. For instance, two pharmacological studies have found that tDCS-induced after-effects take place via the modulation of NDMA glutamatergic receptor activity, which are known to be highly implicated in neuroplasticity (Liebetanz et al., 2002; Nitsche et al., 2003). Furthermore, anodal and cathodal tDCS were found to respectively decrease GABA and glutamate levels (Stagg et al., 2009). Interestingly, anodal tDCS was found to significantly increase the combined glutamate and glutamine levels beneath the stimulating electrode (Clark et al., 2011; Hunter et al., 2015). Finally, in one study combining MRS and fMRI assessment, anodal tDCS over the motor cortex was found to increase motor learning by increasing the resting motor network FC, probably through a decrease in motor cortex GABA levels (Stagg et al., 2014).

Along with the GABA and glutamate changes encountered in MS, a recent work has introduced the dopamine hypothesis in MS fatigue (Dobryakova et al., 2015). Dopamine neurotransmission is also likely involved in NIBS-induced cortical neuroplasticity (Fresnoza et al., 2004, 2014; Nitsche et al., 2006, 2009, 2010; Kuo et al., 2008; Monte-Silva et al., 2009, 2010, 2011; Tanaka et al., 2013).

Thus, tDCS could promote various beneficial synaptic changes which might be helpful in counteracting the deleterious impact of MS on neurotransmission and possibly alleviating fatigue.

### NIBS studies in MS fatigue

To the best of our knowledge, the effects of NIBS on MS fatigue have been evaluated in four studies so far, three of which were based on tDCS use in a double-blind, sham-controlled, crossover design (Ferrucci et al., 2014; Saiote et al., 2014; Tecchio et al., 2014) (Table 6). These studies consisted of two blocks of five consecutive daily sessions of either real or sham tDCS.

**Table 6:** **Studies investing the effects of transcranial direct current stimulation (tDCS) on multiple sclerosis fatigue**.

	**Ferrucci et al., 2014**	**Saiote et al., 2014**	**Tecchio et al., 2014**
Design	Randomized, double-blinded, sham-controlled, crossover study (washout interval: 1 month)	Pseudo-randomized, double-blind, sham-controlled, counterbalanced, crossover study (washout interval: 2 weeks)	Randomized, double-blinded, sham-controlled, counterbalanced crossover study (washout “completed” when the percentage of MFIS difference from baseline becomes < 0.5 of induced effect)
Brain target	Bilateral M1	Left DLPFC	Bilateral whole body S1
Participants	22 RRMS, 3SP	25 RRMS	7 RRMS, 2 PP, 1 SP
Inclusion criteria	Fatigue for at least 6 months defined as MFIS >45; EDSS < 6.5	Fatigue for at least 2 month defined as FSS ≥ 4; EDSS ≤ 6	Experiencing fatigue defined as MFIS >38; EDSS ≤ 3.5
Outcomes	FIS	MFIS, FSS, MS specific FSS	MFIS
Electrodes	Two anodes placed at C3–C4 (10–20 EEG system); the 3rd electrode on the right deltoid	Anode at F3 (10–20 EEG system); cathode placed on the contralateral forehead	Anode on the central sulcus trace; the reference electrode at Oz (10–20 EEG system).
Parameters	1.5 mA, for 15 min	1 mA, for 20 min	1.5 mA, for 15 min
Results	Long-term sustainable effect up to the 3rd week. No correlation between fatigue response and age, disease duration, and EDSS	No significant changes in fatigue scores following stimulation. Changes in perceived fatigue lasting up to day 30. Correlation between lesion load and response to tDCS	Long term sustainable effects up to at least 2 months

*DLPFC, dorsolateral prefrontal cortex; FIS, Fatigue impact scale, FSS, Fatigue Severity Scale; MFIS, Modified Fatigue Impact Scale; M1, primary motor cortex; PP, primary progressive; RRMS, relapsing remitting multiple sclerosis; SP, secondary progressive; S1, primary somatosensory cortex; EDSS, Expanded Disability Status Scale*.

In the first study, Ferruci et al. applied daily sessions of sham or active anodal tDCS over M1 bilaterally, blocks were held apart by at least 1 month (Ferrucci et al., 2014). Two-third of the patients were found to have a significant improvement in their fatigue scores, up to the third week following active tDCS. Sham condition did not result in any significant effects. Interestingly, “responder” patients were found to be younger than their “non-responder” counterparts; no other significant difference was found between both groups in terms of disease duration and severity. Such a difference is quite interesting and could partly explain the results since older patients might be less susceptible to the neuroplasticity processes which could render them less responsive to tDCS (Boggio et al., 2010).

A second study assessed the effects of anodal tDCS over the left DLPFC (Saiote et al., 2014). Fatigue scores improved in about half of the patients following both active and sham tDCS, without any significant difference between the two conditions. The lack of difference between both stimulation conditions could have been resulted from a carry-over effect since stimulation blocks were separated by only 2 weeks which might be too short to exclude interference.

In the third study, following navigation-guided anodal tDCS over the bilateral somatosensory cortices, all patients significantly improved after real but not sham stimulations (Tecchio et al., 2014). Clinical improvement ranged from 2 to 76% and lasted for 8 weeks. Such results can be accounted by some facts. First, concerning stimulation target, the bilateral somatosensory areas are quite and involve the representation areas of the face, upper limbs and lower limbs bilaterally; which might explain its greater efficacy compared to the stimulation of the bilateral hand motor areas or the left DLPFC selected in the abovementioned studies. Second, regarding the study design, the authors stated that the stimulation blocks were not performed in summertime to avoid the induction of fatigue by hot temperatures. In addition, unlike the washout intervals adapted in the other two studies (2 weeks vs. 4 weeks, respectively), a strict washout interval was defined by the authors. In other terms, a patient who completed the first stimulation block was not allowed to have the second one until the MFIS score at the follow-up evaluation satisfies the following condition: $\frac{MFIS\;at\;evaluation\;-MFIS\;preblock1}{MFIS\;preblock1}<0.5\times \frac{MFIS\;postblock1-MFIS\;preblock1}{MFIS\;preblock1}$. This might have minimized any possible carry-over effects that usually add difficulties to the results interpretation. Furthermore, the combination of neuronavigation technique with tDCS could have optimized the outcomes, keeping in mind the superior role of neuronavigation compared to the classical non-navigated interventions (Lefaucheur, 2010).

Apart from tDCS, one iTBS protocol was performed to determine its effects on spasticity, fatigue, and quality of life in 30 RRMS patients: iTBS was applied with or without exercise therapy in a sham-controlled manner (Mori et al., 2011). The stimulation coil was positioned over the M1 leg region contralaterally to the most affected limb, for 10 sessions (protocol duration: 2 weeks; 10 bursts; 600 pulses). iTBS combined with exercise therapy was significantly able to reduce spasticity and fatigue, ameliorating the quality of life. However, iTBS alone could only decrease spasticity, without having any effect on fatigue. Although interesting, such preliminary results need to be replicated and further assessed in future works.

## Conclusion

These data altogether suggest that during the course of the disease, the inflammation along with the demyelination, synaptopathy and neurodegeneration might lead to several structural and functional abnormalities in cortico-subcortical circuits. The latter can take parts in the cortico-striato-thalamo-cortical loop of MS fatigue. Considering MS as a spectrum where the extreme destructive sequels of an MS-attack lie on one pole, and the potential healing and neuroplasticity processes on the other; the clinical manifestations do not seem to exclusively depend on the structural damage, but rather on the balance between restorative and inflammatory/degenerative processes. This vision can be applied to the fatigue loop, the breakdown of which might result in the co-occurrence of the physical, mental, and psychosocial domains of fatigue. Facing the pharmacological limitations in treating MS fatigue, NIBS would pave the way for better therapeutics, especially that tDCS has a promising role in modulating the fatigue loop. In this perspective, optimizing the future protocols would be by acting on several factors. For instance, knowing that tDCS effects on MS fatigue can last for several weeks, extending the washout intervals might clarify the effects of each stimulation blocks by prohibiting the possibility of interference. Furthermore, in the light of discrepancy between the frequently administered fatigue scores and the change in the perceived fatigue on one side, and the complexity of this symptom on the other side, future studies might benefit from adapting better assessment tools, such as the visual analog scale for fatigue. Lastly, combining tDCS protocols with functional imaging would allow for a better understanding of the fatigue loop, which would further help in designing and tuning the ultimate therapeutic plans in MS fatigue.

### Conflict of interest statement

MAC, SSA, NR, RA and JPL declare that the research was conducted in the absence of any commercial or financial relationships that could be construed as a potential conflict of interest. AC gave expert testimony for CSL Behring, Novartis, received grants from Biogen, Novartis, CSL Behring, GE Neuro, Octapharma, and gave lectures for Genzyme.
